# Polarization patterns of light enable geolocalization in oceans

**DOI:** 10.1038/s41377-023-01279-z

**Published:** 2023-09-20

**Authors:** Lihong V. Wang

**Affiliations:** Caltech Optical Imaging Laboratory, Andrew and Peggy Cherng Department of Medical Engineering, Department of Electrical Engineering, California Institute of Technology, 1200 East California Boulevard, MC 138-78, Pasadena, CA 91125 USA

**Keywords:** Imaging and sensing, Optical sensors

## Abstract

The deep ocean, characterized by its immense depths and absence of global positioning system (GPS) functionality, presents considerable challenges for search and rescue missions. Inspired by the geolocalization capabilities of migratory marine animals, Bai et al. present a novel method for underwater geolocalization using the polarization patterns of light in the underwater environment. Emulating a sextant using these underwater polarization patterns, the study determines geolocation in underwater settings. Despite prior skepticism, even in low-visibility waters, these patterns, learned through a deep neural network, provide geolocation accuracies of 55 km at 8 m and 255 km at 50 m. This pioneering approach offers implications for search and rescue and hints at navigation mechanisms in marine animals.

Our enduring fascination with the deep ocean has been further underscored by the recent incident involving the Ocean Gate submersible. This small underwater vehicle, specifically designed to transport tourists to depths of nearly 4 km for a glimpse of the remnants of the Titanic, vanished during its journey. In response, a multinational search and rescue mission was swiftly launched, with time being of the essence in the race to save the stranded passengers. The formidable challenges posed by search and rescue operations in the ocean became immediately apparent. The underwater environment is devoid of global positioning system (GPS) functionality, and the immense pressure exerts significant strain on the capabilities of submersible designs. Additionally, autonomous search robots capable of unrestricted exploration are absent. The entire search and rescue effort relied on tethered vehicles equipped with sonar, which posed limitations on the area that could be covered and hindered the urgency of the search operation.

In the pursuit of untethered underwater geolocalization, Bai et al. propose a solution that leverages the polarization patterns of light observed in the underwater environment^1^. These polarization patterns, found in ocean waters at depths of up to 300 m, are primarily influenced by the positioning of the sun or moon on the horizon. When unpolarized light emanates from these celestial bodies, it penetrates the water and scatters upon encountering suspended particles. Consequently, the underwater polarization patterns serve as a direct indicator of the sun’s or moon’s celestial position. By incorporating precise time and date references, a unique position can be estimated.

The proposed geolocalization method can be likened to a contemporary sextant, a historical navigational tool used for estimating solar heading and elevation angles in GPS-free environments both above water and in aerial navigation. To validate their approach, the authors amassed a vast dataset of underwater polarization videos captured at four distinct locations across the globe, encompassing coastal waters and low-visibility lakes (Fig. 1).

**Fig. 1:**
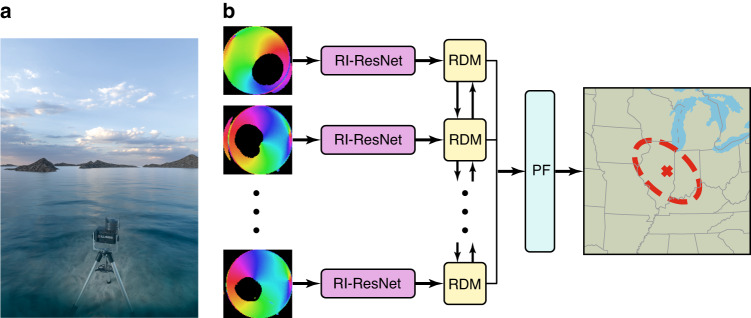
Using underwater polarization patterns to replicate a sextant’s functionality, the study pinpoints geolocation in marine settings. **a** Image sensor capturing underwater polarization. **b** Machine learning model processing polarization images to determine the camera’s longitude and latitude

Contrary to the prevailing belief in the research community that low-visibility waters lack discernible underwater polarization patterns or exhibit solely horizontal polarization, the authors successfully demonstrate that the polarization field surrounding the camera is heterogeneous and reliant on solar polarization. While conventional parametric models for single scattering fail to replicate the observed polarization signatures, a deep neural network proves adept at learning these underwater patterns and aids in estimating the position of the sun. By incorporating temporal information regarding the sun’s trajectory throughout the day, the authors employ a particle filter to determine the camera’s geolocation. This approach achieves a longitudinal accuracy of 55 km at depths of 8 m and 255 km at 50 m of depth.

What is particularly remarkable about this study is that the proposed method also enables geolocalization during nighttime. This represents the first instance in literature where underwater polarization patterns have been recorded and utilized for geolocalization at night, resulting in an impressive accuracy of 1000 km. Notably, this geolocalization accuracy remains consistent regardless of the phase of the moon.

This groundbreaking research on optical-based underwater geolocalization establishes a solid foundation for future advancements in this field. Enhancements to geolocalization accuracy can likely be achieved through the utilization of improved polarization sensors and more sophisticated machine learning algorithms, such as incorporating foundational models. Additionally, enabling geolocalization at greater depths will be crucial for expanding the range of potential applications.

The ultimate question pertains to determining the fundamental limit of geolocalization accuracy and identifying the level of accuracy that suffices for underwater applications. Even with the current method, it can be employed for search and rescue missions, providing rough geolocation initially and subsequently facilitating a more precise search. Furthermore, this research opens up intriguing possibilities for marine animals with polarization vision to utilize similar mechanisms for geolocation and navigation.
